# The Therapeutic Potential of Class I Selective Histone Deacetylase Inhibitors in Ovarian Cancer

**DOI:** 10.3389/fonc.2014.00111

**Published:** 2014-05-20

**Authors:** Dineo Khabele

**Affiliations:** ^1^Department of Obstetrics and Gynecology, Division of Gynecologic Oncology, Vanderbilt University, Nashville, TN, USA; ^2^Vanderbilt-Ingram Cancer Center, Nashville, TN, USA

**Keywords:** histone deacetylases, histone deacetylase inhibitors, epigenetic therapy, ovarian cancer, targeted therapy

## Abstract

Epithelial ovarian cancer remains the deadliest gynecologic malignancy. Despite advances in treatment, new approaches are needed. Histone deacetylases (HDACs) are a family of enzymes that regulate gene expression by removing acetyl groups from lysine residues on histones and non-histone proteins. Inhibition of HDACs with small molecules has led to the development of histone deacetylase inhibitors (HDACi) that are in clinical use, primarily for hematologic malignancies. Although clinical trials with HDACi as single agents in solid tumors have been disappointing, data from independent labs and recent work by our group show that class I selective HDACi have potent anti-tumor effects in pre-clinical models of ovarian cancer. This review summarizes the role of HDACs in ovarian cancer and the potential niche for selective class I HDACi, particularly HDAC3 in ovarian cancer therapy.

## Introduction

Ovarian cancer is the deadliest gynecological malignancy in the United States, with 21,980 new cases and 14,270 deaths estimated for 2014 (1). Epithelial ovarian cancer is classified into two broad subtypes based on biological, histological, and molecular features (2–4). Type I low-grade serous, low-grade endometrioid, clear cell, and mucinous tumors are typically indolent and relatively chemotherapy resistant. Somatic mutations in *KRAS, BRAF, PIK3CA, PTEN, CTNNB1*, and *ARID1A* genes are common in Type I tumors. In contrast, Type II high-grade serous, high-grade endometrioid, carcinosarcoma, and undifferentiated tumors are aggressive, highly proliferative tumors that are relatively chemotherapy sensitive. Type II tumors are genomically unstable with a high degree of copy number alterations, mutations in *TP53*, and alterations in the homologous recombination (HR) DNA damage repair pathway (2–4). HR deficiency confers relative sensitivity to DNA damaging agents such as cisplatin, carboplatin, and more recently PARP inhibitors (2, 5–7). Approximately 70% of epithelial ovarian cancers are Type II high-grade serous tumors (2).

Current treatment for epithelial ovarian cancer entails a combination of cytoreductive surgery and platinum-based chemotherapy (8–12). Platinum-based chemotherapy has extended survival significantly in patients who undergo optimal tumor debulking surgery (13, 14). Despite optimal initial therapy, however, most tumors recur and options for recurrent disease are restricted by few effective drugs and frequent dose-limiting toxicities of traditional cytotoxic drugs (8, 9). Extending the disease-free interval (initial response to platinum therapy) and re-sensitizing tumors to platinum-based drugs (overcoming platinum resistance), while minimizing toxic side effects is an ongoing and urgent clinical dilemma, and new treatment approaches are urgently needed. This review summarizes the role of histone deacetylase inhibitors (HDACi) as epigenetic anti-cancer therapy and evidence that class I selective HDACi, particularly those biased to HDAC3 may be a promising therapeutic strategy for ovarian cancer.

## Histone Deacetylases

Histone deacetylases are a large family of enzymes that deacetylate lysine residues on histones and non-histone proteins (15, 16). Deacetylation of lysine residues of histone tails allows tighter binding of the nucleosome to negatively charged DNA, which results in chromatin compaction. Chromatin compaction is associated with silencing of gene transcription and other functions of genome maintenance such as DNA replication and DNA damage response and repair (16–20). Deacetylation of histones represses the transcription of tumor suppressor genes such as the cyclin-dependent kinase inhibitor, p21 *p21(WAF1/CIP1)*, and the DNA damage repair gene *BRCA1*, and directly or indirectly promotes the expression, activity, or downstream effects of known oncogenes such as *c-MYC* (21), *RAS* (22, 23), and *AKT* (24). Direct deacetylation of non-histone proteins p53, STAT3, c-MYC, α-tubulin, and Hsp90 is implicated in tumorigenesis (15, 25–27).

**Figure 1:**
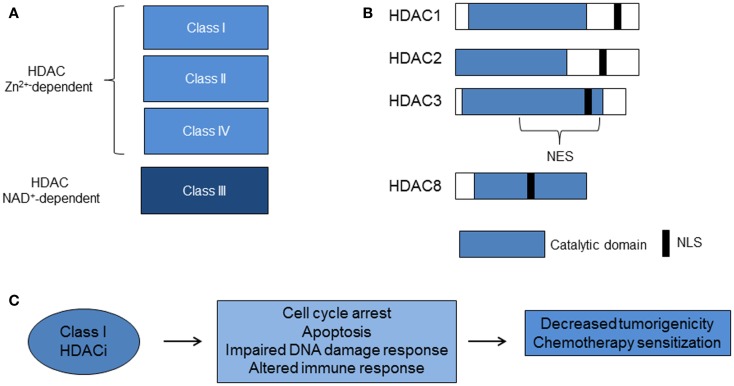
**Histone deacetylases and class I HDACi**. **(A)** Zinc (Zn^2+^)-dependent classes of HDACs. The Class III HDACs are nicotinamide adenine dinucleotide (NAD^+^)-dependent. **(B)** Class I HDACs share more than 50% homology, particularly in the catalytic domain. **(C)** Class I HDACi alter biological pathways that lead to decreased tumorigenicity and chemotherapy sensitization (HDAC, histone deacetylase; HDACi, histone deacetylase inhibitors; NES, nuclear export signal; NLS, nuclear localization signal).

The first mammalian HDAC was discovered by using the small chemical molecule trapoxin as a probe. Trapoxin is a microbially derived cyclotetrapeptide that inhibits histone deacetylation *in vivo* and causes cell cycle arrest in mammalian cells (28). The protein HD1 (HDAC1), similar to the yeast transcriptional regulator Rpd3p/Hda1, was subsequently isolated and cloned. Since then, 18 mammalian HDACs have been identified and are grouped into four classes based on homology to yeast deacetylases. The family of Rpd3/Hda1 are class I (HDAC1, 2, 3, and 8); class IIa (HDAC4, 5, 7, 9); class IIb (HDAC6 and 10); and class IV: HDAC11 (15, 17, 29) (Figure 1). Class I HDACs are ubiquitously expressed, whereas class II and IV HDACs have tissue specificity for smooth muscle, heart, brain, liver, and colon (29). Class III HDACs, related to yeast sirtuins are nicotinamide-dependent enzymes and will not be discussed in this review.

Class I HDACs 1–3 share more than 50% homology, but have distinct structures and cellular functions (15) (Figure 1). HDAC3 lacks the N terminus regions of the other class I HDACs, exists in a distinct multi-protein complex from HDACs 1 and 2, and is associated with N-COR/SMRT co-repressors (15, 16, 30–32). Furthermore, the C terminus of HDAC3 has a unique nuclear export sequence and both nuclear and cytoplasmic localization, which suggests differential function from the other class I HDACs that are confined to the nucleus (33). Knockout mouse models of HDAC1 and HDAC3 enzymes are embryonic lethal and knockout of HDAC2 leads to perinatal death (17). Studies of *in vitro* silencing of HDACs show HDAC1 and 3 siRNA inhibit cell growth and HDAC3 siRNA causes histone hyperacetylation and apoptosis (34–36). These studies point to a critical role for class I HDACs 1–3 in cell growth. HDAC8 does not have known co-repressors and its function remains under investigation.

Aberrant expression of HDACs is implicated in the pathogenesis of malignancies, including solid tumors such as ovarian cancer (35–38). Our group has published that class I HDACs are highly expressed in ovarian cancers (36), and recent work shows elevated class I HDAC expression is associated with poorer survival in certain subtypes of ovarian cancer (37). Because of the pleiotropic pro-tumorigenic effects on cellular proliferation, apoptosis, and DNA damage and aberrant expression of class I HDACs in ovarian cancer, class I HDACi are potentially effective agents for the treatment of ovarian cancer.

## HDAC Inhibitors as Anti-Cancer Drugs

Histone deacetylase inhibitors are a structurally diverse set of chemical compounds traditionally classified into four major categories: hydroxamic acids (e.g., vorinostat formerly SAHA); benzamides (e.g., MS-275); short aliphatic acids (e.g., valproic acid, VPA); and cyclic peptides (e.g., romidepsin or depsipeptide (FK228). Approximately 11 HDACi, including SAHA, MS-275, VPA, and FK228, are in use clinically or are in clinical trials (15, 16, 29, 39, 40). Vorinostat and romidepsin are the only FDA-approved HDACi and are indicated for the treatment of cutaneous T-cell lymphoma (41–44). However, ongoing clinical trials of HDACi in solid tumors, including ovarian cancer (Table 1) (45–48) are underway.

**Table 1:** **Clinical trials of histone deacetylase inhibitors for the treatment of ovarian cancer in the United States**.

Trial/type of study	Treatment/population	Outcomes
NCT00910000 phase IB/II unpublished	Vorinostat, carboplatin, and gemcitabine plus vorinostat maintenance	Terminated
	Recurrent, platinum-sensitive epithelial ovarian, fallopian tube, or peritoneal cancer	Unacceptable toxicity
NCT00976183 phase I/II Mendivil et al. (47)	Paclitaxel, carboplatin, and vorinostat	Terminated
	Primary advanced stage ovarian cancer	Unacceptable toxicity
		3/18 (16.7%) – GI perforation
		Some activity
		7/18 (39%) CR
		2/18 (11.2%) PR
		9/18 (50%) ORR
NCT00993616 phase II Dizon et al. (45)	Belinostat and carboplatin	Terminated due to minimal activity
	Recurrent or persistent platinum-resistant ovarian, fallopian tube, or primary peritoneal cancer	Some activity
		1/27 (3.7%) PR
		12/27 (44.4%) SD
		8/27 (29.6%) PD
		5/27 (18.5%) NA
NCT00421889 phase I/II Dizon et al. (46)	Belinostat, carboplatin, and paclitaxel	Completed
	Previously treated ovarian cancer	No grade 4 toxicities
		Some activity
		3/35 (8.6%) CR
		12/35 (34.2%) PR
		15/35 (43%) ORR
NCT00132067 phase II Modesitt et al. (48)	Vorinostat	Completed
	Recurrent or persistent ovarian or primary peritoneal cancer	Well-tolerated
		Minimal activity
		1/27 (3.7%) PR

*www.clinicaltrials.gov (accessed 4/23/2014)*.

*CR, complete response; PR, partial response; SD, stable disease; PD, progressive disease; ORR, overall response rate*.

Although some HDACi are thought to be non-selective inhibitors, many including FK228 have selective bias toward class I HDACs (49). Our group performed a high-throughput study of a diverse group of HDACi in a panel of ovarian cancer cell lines represented in the NCI 60 panel. We demonstrated that the FK228 is the most potent in reducing cell growth (50). FK228 induced cytotoxic effects, measured by induction of the DNA damage response mark [phosphorylation of histone H2AX (pHAX)], inhibition of cell proliferation and increased cell death. FK228 was isolated from *Chromobacterium violaceum* no. 968, a rare Gram negative bacterium, and approved for the treatment of cutaneous and peripheral T-cell lymphomas (43, 44). The primary mechanism of action of FK228 requires reduction of a characteristic disulfide bond that creates a “warhead” thiol group. The thiol binds to zinc in the catalytic center of both class I and class II HDACs and inhibits HDAC enzymatic activity (51). Based on *in vitro* binding assays, FK228 preferentially inhibits class I HDACs over class II HDACs, with potent biochemical activity against HDAC3 (51). We have gone on to show that class I biased HDACi with similar bicyclic depsipeptide structures, thailandepsin A (TDP-A) and thailandepsin B (TDP-B) discovered from *Burkholderia thailandensis* (52), are as potent as FK228 in ovarian cancer cells (53).

Our group has shown HDACi have potent anti-tumor effects in other ovarian cancer cells with relative resistance to cisplatin (36). These findings suggest a role for HDACi in the treatment of platinum-resistant ovarian tumors. For example, NCI/ADR-Res, an ovarian cancer cell line that is resistant to common cytotoxic agents including cisplatin, is the most sensitive to SAHA in the entire set of ovarian cancer cells represented in the NCI 60 panel (50). HDAC proteins play an important role in DNA damage response and repair, and HDACi are known to reduce the expression of HR associated genes such as BRCA1 and RAD51 (54–57). We have recently shown that SAHA inhibits both BRCA1 and RAD51 in response to DNA damage in ovarian cancer cells (58). This implies a role for HDACi altering HR efficiency as a mechanism for sensitizing ovarian cancer cells to DNA damaging drugs. Whether targeting selective class I HDACs indirectly increases DNA damage, impairs DNA repair, or both is an area of active investigation and has potential therapeutic implications for Type II high-grade serous ovarian cancers.

## Class I HDACi and DNA Damaging Agents

Despite being highly effective *in vitro* and generally well-tolerated *in vivo*, clinical responses to HDACi in solid tumors, including ovarian cancers have been disappointing compared to hematologic malignancies (16, 48). Furthermore, evidence from clinical trials and *in vitro* studies suggest that HDACi are more effective when combined with other anti-tumor agents (16). Table 1 summarizes completed HDACi clinical trials specifically for ovarian cancer in the United States (45–48). In ovarian cancer, single agent trials with HDACi have been disappointing. A Phase II study of single agent vorinostat in platinum refractory recurrent or persistent ovarian cancer showed minimal responses, although it was well-tolerated (48). A Phase II trial of belinostat in women with ovarian cancer, including platinum-resistant disease, showed moderate responses with >50% of the patients with stable disease (59). Interestingly, the best responses were seen in patients with platinum-resistant Type II ovarian cancers in that study. This trend toward improved response in platinum-resistant disease was also observed in a study of belinostat combined with carboplatin and paclitaxel in recurrent ovarian cancer (46). However, in a study specifically for recurrent or persistent ovarian cancer, the combination of belinostat and carboplatin was terminated due to minimal activity (45). The diversity of responses to HDACi in different cell types is not fully understood, but supports observations from our group and others that certain types of cells (e.g., rapidly proliferating cells) are more sensitive than others (e.g., “normal” epithelial cells) to these agents (18, 60). Challenges remain in defining the most appropriate HDACi to combine with other anti-tumor agents.

Histone deacetylase inhibitors have been shown be synergistic with DNA damaging radiation (18, 61–65), suggesting a role for HDACi with DNA damaging chemotherapeutic agents. Combining HDACi with chemotherapeutic drugs that specifically target DNA, such as topoiomerase II inhibitors and cisplatin, enhance the efficacy of these drugs in cancer cells (66–68). Cisplatin or *cis*-diamminedichloroplatinum (II) is one of the first-line standard chemotherapy agents in the treatment of ovarian cancer (9–12). Cisplatin forms covalent platinum-DNA adducts that lead to double strand breaks, DNA damage, and eventual cell death (69, 70). A multi-drug combination of the pan-HDACi belinostat with a DNA methylation inhibitor, decitabine, enhances sensitivity to cisplatin in cisplatin-resistant ovarian cancer cells (71). The potential mechanism of synergy with DNA damaging agents is suggested by published results that selective inhibition of class I HDACs, specifically HDAC3, leads to a defective response to DNA damage, and aberrant histone deposition on chromatin (18, 66). Conditional knock-down of HDAC3 decreases S phase and causes inefficient repair of double strand DNA breaks induced by radiation (18). Because HDAC3 knock-down suppresses cell viability and contributes to DNA damage and disruption of DNA repair (18, 36, 66, 72), we hypothesized that HDACi compounds with HDAC3 bias will be synergistic with DNA damaging agents in ovarian cancer cells and found that FK228, a class I HDACi that potently inhibits HDAC3, enhances the effects of cisplatin *in vitro* and *in vivo* (73).

## Targeting HDAC3 in Ovarian Cancer

Selective class I HDACi targeting HDAC3 is an attractive therapeutic strategy. Our group and others have shown that targeting class I HDACs, particularly HDAC3, inhibits cellular proliferation and directly represses transcription of p21 (35, 36, 74, 75). In acute leukemia, the HDAC3-dependent N-CoR complex is recruited by the oncogenic fusion proteins (76–78). Conditional knock-down of HDAC3 in the liver induces DNA damage, chromosomal instability, and changes in metabolism (18, 20, 72). Inactivation of HDAC3 is sufficient to trigger apoptosis in cycling, non-quiescent murine embryonic fibroblasts, suggesting that HDAC3 could be a therapeutic target in highly proliferative cancer cells (18). HDAC3 is also required for efficient DNA replication in hematopoietic stem and progenitor cells, and required for the passage of hematopoietic stem/progenitor cells through the S phase, for stem cell functions, and for lymphopoiesis (79). The HDAC3 selective inhibitor, RGFP966, causes impaired S phase progression, decreased cell growth, and increased DNA damage associated apoptosis via disruptions in DNA replication in refractory cutaneous T-cell lymphoma (CTCL) (80). These results suggest that HDAC3 and other class I HDACi that enhance DNA damage are effective anti-cancer drugs, but should be used at the lowest doses possible over short periods of time.

## Targeting HDAC3 in the Ovarian Tumor Microenvironment

Observations in ovarian cancer cells and other cancer cell types indicate that selective targeting of HDAC3 may be an attractive therapeutic strategy. Our group and others have shown that inhibiting class I HDACs, particularly HDAC3, inhibits cellular growth and survival, and de-represses p21 transcription leading to increased protein expression in cancer cell lines of diverse origin (35, 36, 74, 75). Class I HDAC inhibition does not induce similar anti-tumor effects in normal ovarian epithelial cell lines (36), consistent with data showing normal, non-transformed cells are spared cytotoxic effects from short-term HDACi treatment (16, 81).

Several studies have indicated that HDAC3 may contribute to inflammatory processes in macrophages during ovarian tumorigenesis, although its role is complex and remains incompletely understood. Ovarian tumorigenesis in the peritoneal cavity involves a complex interplay of signaling and responses between tumor cells and inflammatory cells such as macrophages, T-cells, and dendritic cells (82, 83). The contribution of peritoneal macrophages to the extensive peritoneal tumor implants and malignant ascites characteristic of ovarian cancer is well-recognized (84–86). Thus, therapies designed to alter macrophage function in the ovarian tumor microenvironment are a promising emerging concept reviewed elsewhere (87–89). Our group and others have shown that macrophages are abundant in peritoneal ascites fluid and that ovarian tumors have the ability to polarize macrophages to display tumor-promoting characteristics in a nuclear factor-kappaB (NF-κB)-dependent manner (84, 90), but can be “re-educated” toward an anti-tumor phenotype (84) and thus are a potential target for therapy.

Conditional HDAC3 knock-down in macrophages contributes to the regulation of inflammatory gene expression and function and appears to activate pro-tumorigenic macrophage phenotypes (91, 92). On the other hand, a possible mechanism for an anti-tumor function of HDAC3 in macrophages is direct deacetylation of NF-κB (p65/relA), which is associated with overall termination of the NF-κB transcriptional response (93), but specific activation of transcription of the anti-tumor macrophage cytokine, interleukin-1 (IL-1) (94). Further evidence for the context-dependent role played by HDAC3 in inflammation is that HDAC3 knock-down in macrophages reduces expression of almost half of lipopolysaccharide-induced inflammatory genes (92), but also contributes to transcriptional repression of toll-like receptor activation by deacetylation of p50 (95). These studies indicate that systemic inhibition of HDAC3 may, at least in a specific context, reduce the ability of peritoneal macrophages to mount an anti-tumor response. Therefore, this may represent a possible mechanism by which the efficacy of HDACi in clinical trials is limited. More research is needed to more fully understand HDAC functions in macrophages and inflammatory cells in the peritoneal cavity.

## Future Directions and Limitations

The development of class selective HDACi is ongoing (96). Novel combinations of HDACi with other targeted drugs such as aurora kinase inhibitors are underway (97) and using specific HDACi as hybrid compounds with other anti-cancer drugs is a novel approach (96). For example, targeting HDACs with PI3K inhibitors (96) may have particular relevance for both Type I and Type II ovarian tumors with alterations in the PIK3CA/AKT oncogenic pathway. Finally, hybrid ester-HDACi that selectively trap HDACi in monocytes and macrophages are in development for inflammatory diseases (98) and could be used to target the tumor microenvironment in ovarian cancer.

There are potential limitations to selective HDACi therapy. The effects of class I HDACi on DNA damage and repair pathways suggest that prolonged exposure to these drugs could lead to unacceptable toxicities (47) and secondary malignancies (20). Class I HDACs may also play an oncogenic role depending on the context (20, 99, 100). Further, predictive and prognostic biomarkers of response and toxicity, including potential immune-related toxicities and mechanisms of drug resistance are not known. Finally, the type of combinatory regimen using class I HDACi, dose and sequence of drugs are important considerations that are poorly understood and worthy of further study.

## Conclusion

The limited clinical benefit previously seen with HDACi in ovarian cancer could be explained by a variety of factors including the: (1) phenotypic and molecular features of the tumors; (2) strength and selectivity of HDAC inhibition; and (3) type of combinatory treatment. Substantial pre-clinical evidence shows that class I biased HDACi decrease cell proliferation and increase apoptosis, likely through enhanced DNA damage and decreased DNA repair, in molecularly vulnerable ovarian cancer cells. Combination therapy with other epigenetic drugs such as DNA methyl transferase inhibitors, DNA damaging agents (platinum drugs), and small molecule inhibitors of oncogenic pathways such as the PIK3CA/AKT and NF-κB signaling pathways are potential strategic approaches. Targeting the tumor microenvironment with HDAC3 selective inhibitors is another potentially innovative strategy. A better understanding of the most susceptible ovarian cancer subtypes to target and the most effective HDACi to use in rational combinations with other cancer drugs has the potential to drive novel applications of HDACi in ovarian cancer therapy. Questions about the long-term toxicity of class I HDACi, particularly HDAC3-biased compounds and specific drug combinations remain rich areas for investigation. An ongoing debate in the field is the specificity and selectivity of HDACi as anti-cancer agents. If class I selective HDACi biased to HDAC3 can be designed to improve efficacy in subtypes of ovarian cancer such as in HR deficient ovarian tumors without significantly increasing toxicity, the therapeutic impact could be high.

## Conflict of Interest Statement

Dr. Dineo Khabele discloses that she received a research grant from the Celgene Corporation and is a consultant to Genentech. However, the author is solely responsible for the content.
